# Optimal long-term seed storage conditions for the endangered seagrass *Zostera japonica:* implications for habitat conservation and restoration

**DOI:** 10.1186/s13007-019-0541-6

**Published:** 2019-12-26

**Authors:** Shidong Yue, Yu Zhang, Yi Zhou, Shaochun Xu, Shuai Xu, Xiaomei Zhang, Ruiting Gu

**Affiliations:** 1 CAS Key Laboratory of Marine Ecology and Environmental Sciences, Institute of Oceanology, Chinese Academy of Sciences, Qingdao, 266071 China; 2 Laboratory for Marine Ecology and Environmental Science, Qingdao National Laboratory for Marine Science and Technology, Qingdao, 266071 China; 3 University of Chinese Academy of Sciences, Beijing, 100049 China; 4 Center for Ocean Mega-Science, Chinese Academy of Sciences, Qingdao, 266071 China

**Keywords:** Seagrass, *Zostera japonica*, Seed, Storage condition, Temperature, Salinity, Desiccation sensitivity, Conservation, Restoration

## Abstract

**Background:**

Seagrass meadows are recognized as critical and among the most vulnerable habitats on the planet. The alarming rates of decline in seagrass meadows have attracted the attention globally. There is an urgent need to develop techniques to restore and preserve these vital coastal ecosystems. So far little work has been done to develop effective long-term storage method for seagrass seeds. The seagrass *Zostera japonica* Asch. & Graebn is an endangered species in its native range. Here we utilized combinations of different storage times, salinities, and temperature to determine the most appropriate conditions for optimal seed storage.

**Results:**

*Zostera japonica* seeds were strongly desiccation sensitive, with a complete loss of viability after 24 h of desiccation. Therefore, long periods of exposure to air should be avoided to minimize seed mortality. In addition, *Z. japonica* seeds could not endure freezing conditions such as – 5 °C. However, our results indicated that reduced storage temperature to 0 °C could effectively prolong the duration of dormancy of *Z. japonica* seeds. Seeds stored at 0 °C under a salinity of 40–60 psu showed relatively low seed loss, high seed vigor and fast seed germination, suggesting these to be optimal seed storage conditions. For example, after storage for 540 days (ca. 600 days since the seed collection from reproductive shoots in early October, 2016) at 0 °C under a salinity of 50 psu, seeds still had a considerable vigor, i.e. 57.8 ± 16.8%.

**Conclusion:**

Our experiments demonstrated that seeds stored at 0 °C under a salinity of 40–60 psu could effectively prolong the duration of dormancy of *Z. japonica* seeds. The proposed technique is a simple and effective long-term storage method for *Z. japonica* seeds, which can then be used to aid future conservation, restoration and management of these sensitive and ecologically important habitat formers. The findings may also serve as useful reference for seed storage of other threatened seagrass species and facilitate their ex situ conservation and habitat restoration.

## Background

Seagrass meadows are recognized as critical and threatened marine habitats around the world. Seagrasses are a unique group of submerged marine angiosperms distributed along the temperate and tropical coastlines of the world [15, 50]. They evolved from terrestrial plants some 100 million years ago and now colonize coastal waters. As important ecosystem engineers and habitat formers, they form the basis of one of the most widespread and productive coastal ecosystems in the world, providing food, essential habitats, and nurseries for a variety of marine organisms [3, 4, 12, 18, 20, 25, 37, 54], as well as reducing exposure to bacterial pathogens of humans, fish, and invertebrates [31]. However, seagrass meadows are disappearing at an alarming rate worldwide and face anthropogenic and natural threats globally [40, 59, 39, 51, 52, 57]. Thus, effective management and active restoration programs are becoming increasingly important [13, 34, 56, 58].

Attempts have been made to repair damaged seagrass meadows [28, 38, 42, 44, 70]. Seeds have an important role in the reproduction of seagrasses, such as *Zostera marina* and *Cymodocea nodosa*, and using seeds to repair damaged meadows has resulted in faster recovery than transplanting plants [42]. It is also less harmful to the original seagrass meadow, can maintain the genetic diversity of seagrass populations, and can reduce the cost of restoration. However, such restoration is limited by seed collection and processing, seed preservation, and the low germination rate of seedlings [43]. Therefore, determining the optimal preservation conditions for seagrass seeds is important to promote the successful regeneration of seagrass meadows. It is well known that seed storage can be employed in greenhouse seedling propagation and is a valuable method of ex situ conservation and habitat restoration.

Salinity is generally considered to be the most important factor affecting the germination of seagrass seeds [41]. Salinity has species-specific effects on seagrass seed germination, whereas temperature has a greater impact on seagrass seed germination, but this is usually limited by the level of salinity. Both are important factors that affect the preservation of seagrass seeds. *Z. marina* seeds are unable to resist desiccation and, therefore, must be kept moist [45]. Suitable environmental conditions can prolong the duration of dormancy of seagrass seeds. So far, seagrass seeds are generally stored in natural seawater at 4–7 °C [1, 14, 16, 26, 27, 29, 46, 62]. *Z. capricorni* seeds can be stored for 50 days under low temperature (5–10 °C) conditions [11], and the germination time of *Phyllospadix torreyi* seeds can also be postponed by 83 days at 4 °C under dark storage conditions [48]. *Z. marina* seeds preserved in seawater and at room temperature can survive for 8 months [10]. Dooley et al. [14] reported that *Z. marina* seeds could survive for several years at 5 °C. However, Kaldy et al. [26] showed a cumulative seed loss rate of >52% after 14 months of storage at 4 °C. Thus, seed loss during storage is an important factor that should be considered when evaluating seed preservation methods.

Aerobic or anaerobic conditions are important variables affecting seed germination of aquatic plants, including *Zostera* species [7, 47]. Probert and Brenchley [47] reported that seeds germinated more rapidly and to a higher final percentage under anaerobic conditions compared with aerobic conditions. Thus, to avoid seed germination during storage, it is suggested that aerobic conditions should be used for long-term seed storage.

*Zostera japonica* Asch. & Graebn. (Alismatales: Zosteraceae), is an intertidal seagrass species that is native to Asia from the temperate area of Sakhalin, Russia to subtropical southern Vietnam [15]. It is declining and recognized as endangered species in many parts of Asia, including Japan [2, 21], Korea [33] and China [68]. This species has undergone a severe decline in China, and some populations have almost, if not entirely, disappeared [23, 35, 36, 67, 68]. By contrast, *Z. japonica* has been successfully introduced to the coastlines of British Columbia (Canada), Washington, Oregon, and North California (USA), where it has become established [49]. The decline of this species in its native range has been attributed to anthropogenic disturbances such as coastal development, river channel improvements, aquaculture and harvesting activities [2, 23, 32, 33]. Therefore, conservation and restoration efforts are required urgently for *Z. japonica* in its native range.

Few studies have focused on the optimal conditions for the preservation of *Z. japonica* seeds. Japanese researchers reported that *Z. japonica* seeds maintained at 4 °C in seawater showed better preservation compared with seeds stored at other temperatures [29], however, this temperature still has a relatively high seed loss during storage due to germination of seeds [26, 29], therefore the storage condition is not optimal for long-term *Z. japonica* seed storage. Therefore, there is a need to investigate further the preservation effects on *Z. japonica* seeds at more effective temperatures, combined with different levels of salinity, given the known relevance of this environmental factor on seed viability. Thus, in this study, we investigated the hypotheses that: (1) completely dry conditions would be detrimental to the long-term preservation of *Z. japonica* seeds; and that (2) *Z. japonica* seeds would be better preserved at lower temperature under higher salinity. Therefore, the aims of this study were to identify the optimal salinity and temperature conditions for long-term seed storage, which could facilitate the conservation and restoration of seagrass beds.

## Materials and methods

### Site description and seed collection

In early October 2016, a large number of reproductive branches with immature *Z. japonica* seeds were collected from Swan Lake, Rongcheng City, Shandong Province (37°35′ N, 122°57′ E) and stored in the laboratory until use. The branches were maintained in a recycled water aquarium (natural seawater, 15–20 °C), until the end of December 2016. At this point, the reproductive branches had degraded and the ripe seeds were removed and saved for further.

To analyze the moisture content (MC_0_; %) of *Z. japonica* seeds, 150 seeds were randomly selected and divided to three replicates. Seeds were spread on soft paper towels to remove adherent water, and the wet weight (M_W_) of seeds was measured. Seeds were then placed in a drying oven for 1 h (130 °C) and cooled at 30% relative humidity (International Seed Testing Association [ISTA], [24]). The seed dry weight (M_D_) was then measured. MC_0_ was calculated using Eq. 1:

1 $$MC_{0} = \frac{{M_{W} - M_{D} }}{{M_{W} }} \times 100\%$$

## Seed storage in dry conditions

Four drying times (1 day, 3 days, 7 days, 15 days) were selected, with three replicates in each experimental group, each containing 50 seeds of the same color. The surface water of the seeds was removed with soft paper towels, and the wet weight (M_W_) of seeds was measured. The test seeds were then dried at a humidity of 33 ± 10% and at a temperature of 20 °C. After drying exposure at each duration, the seed weight (M_S_) was measured.

The moisture content (MC; %) of the seeds under each desiccation duration was calculated using Eq. 2:

2 $$MC = \frac{{M_{S} - M_{W} (1 - MC_{0} )}}{{M_{S} }} \times 100\%$$

where *MC*_*0*_ represents initial seed moisture content (%) before desiccation treatment, as calculated in Eq. 1.

To determine the *Z. japonica* seed vigor [65], each replicate was placed in a 150-mL beaker containing artificial seawater (salinity 5 psu), and the beaker was sealed with a sealer and transferred to a 15 °C light incubator (GXZ-280D, Ningbo Jiangnan Instrument Factory) covered with a shading cloth. The water was changed every 5 days for a total of 30 days, and the number of seeds that had germinated was recorded. Any seeds that had germinated were removed from each beaker. The seawater used for each water change was preheated for 4 h before being put in the beaker. The seed germination rate was expressed as the cumulative germination rate over the 30-day experimental period [65].

## Seed storage in wet conditions

Two different temperatures (0 °C and 4 °C) and six different salinity conditions (20, 30, 40, 50, 60, and 70 psu) were established. The control group was maintained at room temperature, with a salinity level of 30. Before the test, the seawater in each beaker was precooled for 4 h.

Artificial seawater (1 L) of the relevant salinity was added to a 1 L rounded glass bottle, to which was then added 1000 *Z. japonica* seeds. The bottle was sealed, covered with a cloth, and placed in either a 0 °C or a 4 °C low-temperature incubator (KRC-150CA type, Shanghai Qi Xin Scientific Instrument Co., Ltd.). The seawater was changed every 30 days to avoid the occurrence of anaerobic environment, following the protocol described above. The trial began on December 31, 2016 and ended on June 24, 2018, with a duration lasting 540 days.

During the storage period, germinated and decomposed seeds were regarded as seed losses, which were recorded every 30 days. The percentage seed loss was the proportion of accumulated seed losses relative to the total number of *Z. japonica* seeds in the treatment, as follows:

$$\text{Seed loss} = \frac{{n}_{i}}{{N}_{i}} \times 100\%$$

where *n*_*i*_ represents the accumulated number of seeds lost, *N*_*i*_ represents the total number of seeds in the treatment, and *i* represents the treatment number.

After storage for 60, 120, 210, 300, 360, and 540 days, three replicates of seeds (50 seeds per replicate) were obtained for vigor test. The vigor of nongerminated seeds was tested as described above. As regards storage for 540 days, we chose only one potential storage condition at 0 °C under a salinity of 50 psu to test seed vigor, based on the test results of seed vigor after storage within 360 days.

## Seed storage at − 5 °C

To examine the effect of storage temperature at − 5 °C on seed viability, three storing times (1 day, 3 days, 7 days) under dry and wet conditions were selected, with four replicates (50 seeds per replicate) in each experimental group. The vigor of seeds in these treatments were tested as described above.

## Statistical analyses

One-way analysis of variance (ANOVA) was employed to compare the effects of the dry exposure durations in the short-term dry exposure experiment. The seed moisture contents after storing in dry conditions were also analyzed using one-way ANOVA. Two-way ANOVA was employed to compare the effects of storage temperatures and salinities in the wet storage experiment. When the interaction was significant, a simple effect test (a one-way ANOVA and Tukey’s multiple comparisons or an Independent-Samples T Test) was conducted when the effects of storage temperatures and salinities were both significant (*p* < 0.05) [66]. Two-way ANOVA was also employed to compare the effects of storage times and conditions in the  −  5 °C storage experiment. When the interaction was significant, a simple effect test (a one-way ANOVA and Tukey’s multiple comparisons or an Independent-Samples T Test) was conducted when the effects of storage times and conditions were both significant (*p* < 0.05) [66]. Statistical analyses were conducted using SPSS 17.0.

## Results

### Short-term storage in dry conditions

The dry exposure duration had significant effects on seed survival. None of the seeds stored in dry conditions germinated, regardless of their treatment group. The initial seed moisture content (MC_0_) before desiccation treatment is 51.5 ± 0.7%. At different drying times, the moisture content of the *Z. japonica* seeds showed no significant difference (*p* > 0.05, Table 1).

**Table 1:** Moisture content of *Z. japonica* seeds maintained in dry conditions for different lengths of time

Dry exposure duration (days)	Moisture content (%)
1	10.0 ± 0.8^a^
3	8.0 ± 13.6^a^
7	− 1.8 ± 2.0^a^
15	− 3.0 ± 20.8^a^

Different letters indicate significant differences at *p* < 0.05 (mean ± SE)

## Long-term storage in wet conditions

**Fig. 1:**
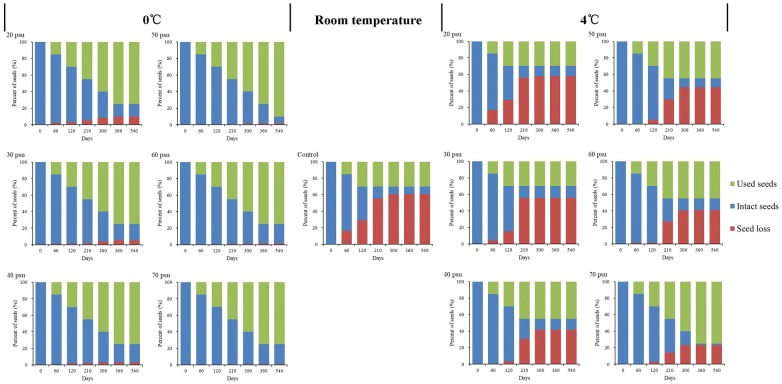
*Zostera japonica* seed composition under different salinity and temperature conditions in long-term wet storage experiments. Used seeds: seeds used for germination experiments; Intact seeds: hard, black seeds; seed loss: seeds that had decomposed or germinated during storage. The control group comprised seeds kept at 30 psu and room temperature (10–25 °C)

The control seeds germinated rapidly at a salinity of 30 psu and under variable room temperature (10–25 °C) conditions, where the percentage seed loss was 60.8% (Fig. 1). There were significant differences between 0 and 4 °C temperatures for long-term seed storage. There was a high seed loss percentage at 4 °C, with the highest seed loss occurring at 4 °C under a salinity of 20 psu, i.e. 57.8% after storage for 360 days. By contrast, all the salinity treatments at 0 °C obtained lower seed loss percentages. Compared with the seeds stored at 4 °C, the seeds stored at 0 °C were also less likely to decay duo to infection by bacteria.

**Fig. 2:**
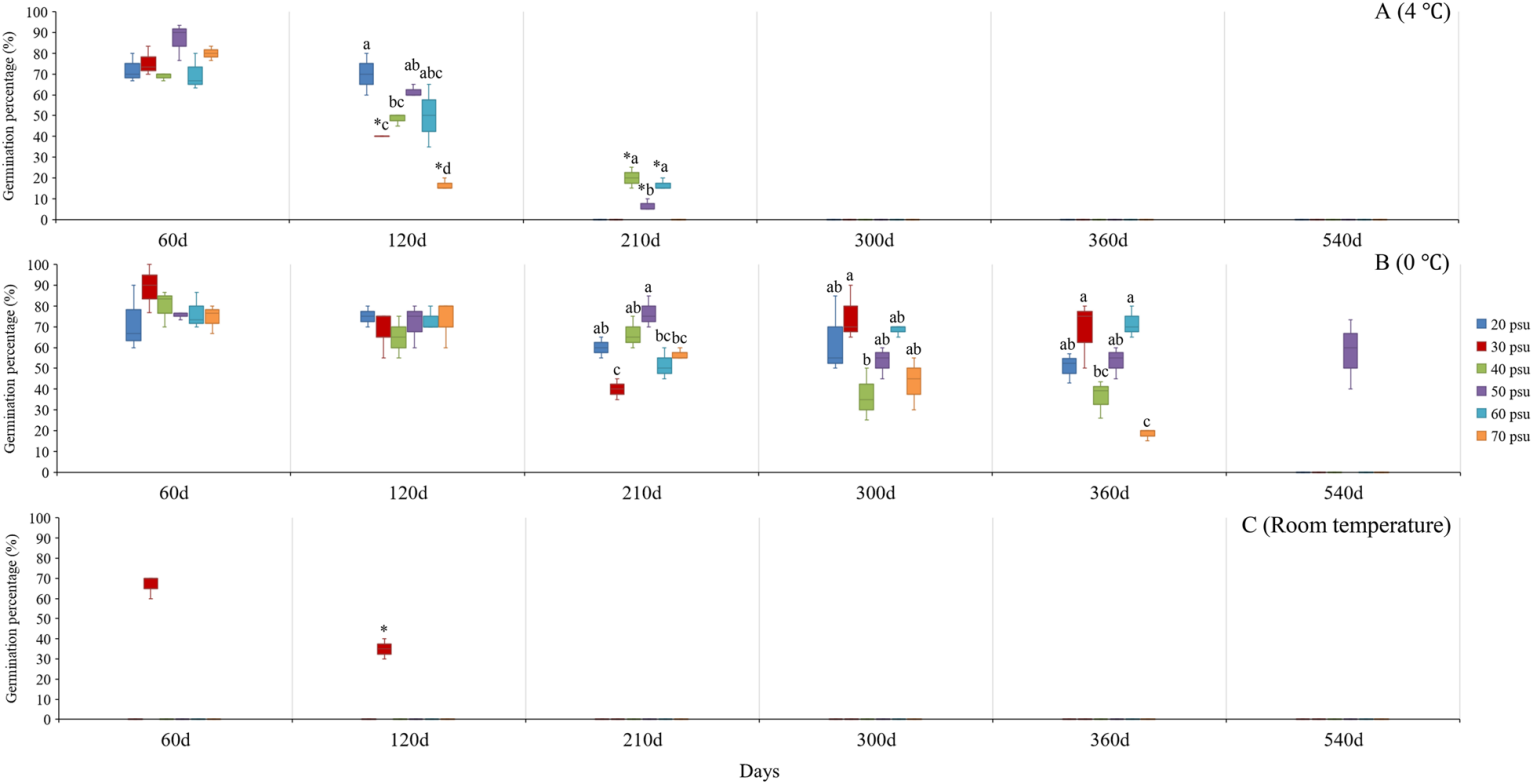
*Zostera japonica* seed vigor after storage under different salinities and temperatures. * indicates significant difference between different temperatures (0 °C, 4 °C and room temperature) under the same salinity treatment and the same storage time. Different letters indicate significant difference between different salinities under the same temperature treatment and the same storage time

All the storage treatments had greater seed vigor levels than the control groups (seed storage at salinity 30 psu and room temperature; Fig. 2). After storage for 60 days, salinity had no significant effect on seed vigor, with seed vigor in all treatments ranging from 66.7 ± 5.8% to 88.9 ± 11.7%. However, seed vigor was significantly different at different storage temperatures (*p* < 0.05, Table 2). And the seed vigor gradually increased with the decrease in storage temperature.

**Table 2:** Statistical differences in the effects of storage temperature and salinity on the number of germinated seeds after storage for 60 days

Variable	df	Sum square	Mean square	*F*-value	*P*
Two-way ANOVA					
Model	12	1551.850	129.321	1.886	0.085
Storage temperature	2	536.167	268.084	3.910	0.033
Storage salinity	5	519.291	103.858	1.515	0.220
Storage temperature × storage salinity	5	697.663	139.533	2.035	0.107

After storage for 120 days, salinity and temperature had significant interactive effects on seed vigor (*p* < 0.001, Table 3). There was no significant effect of salinity on seed vigor at 0 °C. However, there was a significant effect of salinity on seed vigor at 4 °C (*p* < .001, Table 3). The highest seed vigor (70.0 ± 0.0%) among all treatments was recorded at 4 °C under a salinity of 20 psu. Moreover, seed vigor was below 20% at 4 °C under a salinity of 70 psu. Under the same salinity, all treatments at 0 °C had the highest seed vigor (Fig. 2).

**Table 3:** Statistical differences in the effects of storage temperature and salinity on the number of germinated seeds after storage for 120 days

Variable	df	Sum square	Mean square	*F*(or *T*)value	*p*
Two-way ANOVA					
Model	12	11,926.923	993.910	14.225	< 0.001
Storage temperature	2	5634.722	2817.361	40.322	< 0.001
Storage salinity	5	2830.556	566.111	8.102	< 0.001
Storage temperature × storage salinity	5	2541.667	508.333	7.275	< 0.001
One-way ANOVA					
Storage temperature (°C)					
0	5	211.111	42.222	0.475	0.788
4	5	5161.111	1032.222	17.695	< 0.001
Storage salinity (psu)					
30	2	1938.889	969.444	18.368	0.003
Independent-samples T Test					
Storage salinity (psu)					
20	4			0.775	0.482
40	4			2.774	0.05
50	4			1.604	0.184
60	4			2.514	0.066
70	4			8.246	0.001

Seed loss in each treatment increased gradually with the increase in storage time. When the storage time elongated to 210 days, seed loss in the control and 4 °C under a salinity of 20–30 psu had exceeded 55%. There was a significant effect of salinity on seed vigor at 0 °C (*p* < 0.001, Table 4). The highest seed vigor (76.7 ± 7.6%) among all treatments was recorded at 0 °C with a salinity of 50 psu. Under the same salinity, all treatments at 0 °C had the highest seed vigor (Fig. 2).

**Table 4:** Statistical differences in the effects of storage temperature and salinity on the number of germinated seeds after storage for 210 days

Variable	df	Sum square	Mean square	*F*(or *T*) value	*p*
One-way ANOVA					
Storage temperature (°C)					
0	5	2373.611	474.722	12.207	< 0.001
4	3	758.333	252.778	24.267	< 0.001
Independent-samples T test					
Storage salinity (psu)					
40	4			8.854	0.001
50	4			14.849	< 0.001
60	4			7.425	0.002
70	4			34.000	< 0.001

Due to great seed loss through seed germination and seed decay, in most storage treatments at 4 °C there were no enough seeds left for vigor test after storage for 300 days; Only in one treatment (70 psu) seed loss less than 40%. There was a significant effect of salinity on seed vigor at 0 °C (*p* < 0.05, Table 5). The highest seed vigor (75.0 ± 3.2%) among all treatments was recorded at 0 °C with a salinity of 30 psu. In contrast, no viable seeds were observed at 4 °C under a salinity of 70 psu. The results of seed vigor test after storage for 360 days were similar (Table 6). After storage for 540 days, seeds storage at 0 °C under a salinity of 50 psu still had a considerably high vigor, i.e. 57.8 ± 16.8%.

**Table 5:** Statistical differences in the effects of storage temperature and salinity on the number of germinated seeds after storage for 300 days

Variable	df	Sum square	Mean square	*F*(or *T*) value	*p*
One-way ANOVA					
Storage temperature (°C)					
0	5	3316.667	663.333	4.342	0.017
Independent-samples T test					
Storage salinity (psu)					
70	4			5.965	0.004

**Table 6:** Statistical differences in the effects of storage temperature and salinity on the number of germinated seeds after storage for 360 days

Variable	df	Sum square	Mean square	*F*(or *T*) value	*p*
One-way ANOVA					
Storage temperature (°C)					
0	5	6027.972	1205.594	13.977	< 0.001
Independent-samples T test					
Storage salinity (psu)					
70	4			11.000	< 0.001

## Seed storage at − 5 °C

Storage time and condition had significant interactive effects on the vigor of seeds (F = 13.413, df1 = 2, df2 = 18, *p* < 0.001). Storage at − 5 °C dramatically reduced the seed vigor (Table 7). Seed vigor after storage for 1 day was reduced to as low as 5.4 ± 4.1% and 31.0 ± 13.2% under wet and dry conditions, respectively; and no viable seeds were observed after storage for 7 days.

**Table 7:** Vigor of *Z. japonica* seeds maintained at − 5 °C

Storage time (days)	Storage condition	Seed germination percentage (%)
1	Dry	31.3 ± 13.8^*a^
	Seawater	5.0 ± 4.1^a^
3	Dry	1.3 ± 2.5^b^
	Seawater	6.3 ± 6.3^a^
7	Dry	0.0 ± 0.0^b^
	Seawater	0.0 ± 0.0^a^

^*^ Indicates significant difference between two different storage conditions (dry and under seawater) under the same storage time

Different letters indicate significant difference between different storage times under the same storage conditions

## Discussion

In this study, we tested the impact of different storage conditions on the viability of seeds of the endangered seagrass *Zostera japonica*. We found that *Z. japonica* seeds were strongly desiccation sensitive and could not endure freezing conditions such as − 5 °C. We observed that *Z. japonica* seeds could tolerate relatively low temperature and high salinity conditions, indicating a potential method for long-term wet storage of seeds. We first showed that 0 °C was an optimal temperature for long-term wet storage of *Z. japonica* seeds; and we proposed that a temperature of 0 °C under a salinity of 40–60 psu was the optimal conditions for long-term wet storage of *Z. japonica* seeds. The findings could be used to improve the long-term storage conditions for *Z. japonica* seeds, since they provide a useful reference for the establishment of seed banks of *Z. japonica* seeds that could be used to restore degraded seagrass beds.

Currently, the main methods for monitoring viability in seed banks include germination tests, 2,3,5-triphenyltetrazolium hydrochloride dyeing, and electric conductivity measurements [61, 63]. The optimal water temperature range for seed germination is in the range 15–20 °C [2]. In addition, low salinity (5 psu) can break the physiological dormancy of seagrass seeds [16, 17, 19]. Thus, the conditions (15 °C and 5 psu) used in our germination tests were optimal for seed germination. At the beginning of our experiments, the seed germination rate under optimal conditions was ~ 80%, indicating that these germination conditions could easily break the physiological dormancy of seagrass seeds. Dooley et al. [14] used both germination and viability staining to determine the viability of 1-year-old *Z. marina* seeds. Their results indicated that germination rates of *Z. marina* were consistent in trend but somewhat lower than viability rates by staining, with seed viability being 77% by staining and 68% by germination (i.e. a difference of <15%), indicating that the viability values determined by the two methods were relatively comparable. Thus, it is suggested that, in the current study, seed germination rates under optimal conditions after 30 days could well reflect seed vigor.

Results from the short-term drying experiment showed that *Z. japonica* seeds were strongly desiccation sensitive, losing their viability completely after desiccating for 24 h. This is similar to *Z. marina* seeds by Pan et al. [45] who reported that *Z. marina* L. seeds lost their viability after being dried for 24 h. Thus, long periods of exposure to air should be avoided to minimize the mortality of seeds to be used in restoration projects. Seed viability in *Ruppia sinensis* is negatively correlated with seed moisture content [17], also, Xu et al. [62] observes the same correlation in *Z. marina*. Seed desiccation tolerance is generally divided into three broad categories [55]: desiccation tolerant (orthodox), intermediate, and desiccation sensitive (recalcitrant). The trait has important implications for species conservation and restoration, as desiccation-sensitive seeds cannot be stored using traditional seed banking techniques. Globally, most flowering plants including Alismatales produce desiccation-tolerant seeds [60]. However, it is suggested that the majority of seagrass seeds should be desiccation sensitive, although little is known about the seed ecology of these species, so far, only *Ruppia* species such as *R. maritima* and *R. sinensis* are possibly intermediate in seed desiccation tolerance, and their seeds can survive dry conditions for some months [9, 17].

Seed dormancy refers to the phenomenon whereby seeds are unable to germinate even under appropriate environmental conditions (light, temperature, etc.) [5]. The advantage of this phenomenon is that it protects seeds from germinating under adverse conditions. Thus, it would be useful to maintain the dormancy of seagrass seeds until environmental conditions are suitable for their germination [41]. *Zosteraceae* seeds have a hard shell with distinct dormancy features [30]. Many factors affect the dormancy of seeds, with the most important factors generally considered to be salinity [30] and temperature [17, 27]. High salinity affects seed dormancy via osmotic or ionic interactions [7]. It is known that reduced temperature (e.g., 4–7 °C) can prolong the dormancy of seagrass seeds; moreover, under low temperatures, the growth of fungi and other microorganisms is inhibited and the rate of seed decay decreases. In addition, seed dormancy may also differ between different geographic populations, even under the similar surrounding salinity and temperature conditions [64].

Reduced salinity has been reported to increase the germination rate of *Z. japonica* seeds [26, 65]. The highest germination rate of *Z. noltii* seeds occurred at 30 °C and a salinity level of 1 psu, whereas the germination rate of *N. nobilis* decreased with decreasing temperature and salinity [22]. More than 90% of *Cymodocea nodosa* seeds germinated within 10 days under both high temperature and low salinity conditions [8], which is consistent with the results obtained in the current study: at the same temperature, the higher the salinity, the lower the rate of seed loss.

Suitable environmental temperature conditions can prolong the dormancy of seagrass seeds. The current results showed that the vigor of seagrass seeds decreased gradually with increasing temperature, which was consistent with the results of Conacher et al. [11] and Reed et al. [48]. Generally, seagrass seeds are stored in natural seawater at 4–7 °C [1, 14, 16, 26, 27, 29, 46, 62], however, this temperature still has a relatively high seed loss during storage due to germination of seeds and seed decay [26, 29]. According to Kaldy et al. [26], the cumulative *Z. japonica* seed loss rate was > 52% after 14 months of storage at 4 °C and 34 psu. This is consistent with our experimental results; after 14 months of storage at 4 °C and 30 psu, our cumulative seed loss rate was 54%. Also, Dooley et al. [14] reported that *Z. marina* seeds can survive for several years at 5 °C; however, seed loss during long-term storage at 5 °C was not mentioned. Thus, it is suggested that seed loss during storage should be considered when evaluating seed conservation methods. In the present study, both seed loss and seed vigor were used as indicators for evaluating the preservation methods. Our results showed that 0 °C was more conducive to the dormancy of *Z. japonica* seeds. Gu et al. [17] reported that *R. sinensis* seeds maintain vigor for a week at − 10 °C; by contrast, our results showed that *Z. japonica* seeds lost viability dramatically at − 5 °C, indicating − 5 °C is not a suitable temperature for long-term storage. Unlike some seagrass species such as *Enhalus acoroides* seeds that cannot endure low temperature even for a short period, the preservation effectiveness for *Z. japonica* seeds was most obvious at 0 °C, suggesting that is the ideal preservation temperature. In addition, the results of the current study showed that salinity affected seed vigor, and seed germination rate in optimal germination conditions was faster for seeds stored under higher salinity. Therefore, salinity is also a factor that affects the preservation of *Z. japonica* seeds.

Kaldy et al. [26] reported that *Z. japonica* seeds can remain viable in storage for at least 26 months at 4 °C, suggesting that they may be capable of developing a persistent seed bank in situ. This was supported by Bigley [6] who found that 75% of "old seed" (obtained from the top 20 cm of sediment) was still viable after almost a year in storage at 5 °C and 27 psu. However, in situ seed banks of *Z. japonica* in China and Korea could only be maintained for ~8 months [53, 69]. Especially, Xu et al. [64] reported that *Z. marina* seeds in Swan Lake had a 9-month dormancy period, whereas in HuiQuan Bay, only a 4-month dormancy period. Thus, it is suggested that the seeds from different geographical populations may have different dormancy periods. In the present study, the viability of seeds after 210 days of storage at 4 °C was reduced to ~20%, which was lower than that reported by Kaldy et al. [26]; this might be because of the different storage conditions used. Seeds are susceptible to microbial infection (fungi or bacteria) at 4 °C under static water conditions. In Kaldy et al.’s study, all seeds were stored at 4 °C and 34 psu until initiation of germination tests, and seawater volume and salinity were adjusted monthly as needed; the cumulative seed loss rate was 9% after 6 months. In the present study, 1000 seeds were placed in 1 L seawater, and the cumulative seed loss rate was 15.3% after 6 months of conserved seed storage at 4 °C and 30 psu. Thus, in our study, after 210 days of storage at 4 °C, seeds might be more prone to microbial infection (fungi or bacteria), which could explain the relatively low vigor of these seeds.

## Conclusion

In conclusion, this study indicated that *Z. japonica* seeds were strongly desiccation sensitive, and long-term seed storage could only be practiced in wet conditions. Reduced storage temperature and increased salinity could greatly prolong the duration of dormancy of *Z. japonica* seeds. In this study, we developed a feasible long-term seagrass seed storage method. The ideal storage conditions for *Z. japonica* seeds were storage at 0 °C under a salinity of 40–60 psu; such conditions resulted in a reduced rate of seed loss (≤ 10%), higher seed vigor, and faster seed germination. These results provide insights that can be used to develop seed banks and storage facilities for maintaining a supply of *Z. japonica* seeds that can be used for the large-scale restoration of damaged seagrass meadows around the world. The findings may also serve as useful reference for seed storage of other threatened seagrass species and facilitate their ex situ conservation and habitat restoration.

## Data Availability

Data is available on request to the authors.

## Abbreviations

MC: moisture content of seeds under each desiccation duration
MC_0_: initial moisture content of seeds before desiccation treatment
